# An Opinion Facilitator for Online News Media

**DOI:** 10.3389/fdata.2021.695667

**Published:** 2021-07-06

**Authors:** Tom Willaert, Paul Van Eecke, Jeroen Van Soest, Katrien Beuls

**Affiliations:** Artificial Intelligence Laboratory, Vrije Universiteit Brussel, Brussels, Belgium

**Keywords:** news websites, semantic frames, climate change, opinion facilitation, debate

## Abstract

With more and more voices and opinions entering the public domain, a key challenge facing journalists and editors is maximizing the context of the information that is presented on news websites. In this paper, we argue that systems for exposing readers to the many aspects of societal debates should be grounded in methods and tools that can provide a fine-grained understanding of these debates. The present article thereby explores the conceptual transition from opinion observation to opinion facilitation by introducing and discussing the Penelope opinion facilitator: a proof-of-concept reading instrument for online news media that operationalizes emerging methods for the computational analysis of cultural conflict developed in the context of the H2020 ODYCCEUS project. It will be demonstrated how these methods can be combined into an instrument that complements the reading experience of the news website *The Guardian* by automatically interlinking news articles on the level of semantic frames. In linguistic theory, semantic frames are defined as coherent structures of related concepts. We thereby zoom in on instances of the “causation” frame, such as “climate change causes global warming,” and illustrate how a reading instrument that links articles based on such frames might reconfigure our readings of climate news coverage, with specific attention to the case of global warming controversies. Finally, we relate our findings to the context of the development of computational social science, and discuss pathways for the evaluation of the instrument, as well as for the future upscaling of qualitative analyses and close readings.

## Introduction

In our present society, news websites play a pivotal role in documenting as well as shaping societal debates. However, along with other innovations such as social media, news websites have been affected by the rapid expansion of the digital realm, which has led to information overload, fragmentation and polarization (Rusbridger, 2018, pp. xx-xxi). As more and more voices and perspectives enter the public domain through different (social) media, journalists and editors face the challenge of presenting information to their readers in a way that does justice to the many aspects of societal debates, and that maximizes the content and opinion diversity of online news (INMA, 2018). In response to this challenge, many news websites have adopted data-driven systems to identify and expose their readership to those perspectives and pieces of content deemed relevant (Hendrickx et al., 2021), and the technological development of recommender systems for the news industry has grown into an active area of research (Portugal et al., 2018; Da’u and Salim, 2020; Fengh et al., 2020). As has been revealed on the basis of various algorithmic auditing efforts, however, systems for content curation and news personalization can be marked by problems including bias reinforcement, and their inner workings might remain opaque to the end users of the recommendations (Sandvig et al., 2014; Elami et al., 2015).

Acknowledging these pitfalls, we argue that any reading instrument for online news content that aims to automatically expose a readership to the multi-faceted nature of cultural or societal conflicts, should be grounded in an actual understanding of the complexities of these debates. Therefore, these systems should be built around methods for observing and representing cultural conflict in fine-grained manners. The development of such tools and techniques for observing cultural conflict on the basis of digital (textual) data is central to the H2020 Opinion Dynamics and Cultural Conflict in European Space (ODYCCEUS project) (Odycceus, n.d.). This interdisciplinary project explores a range of text mining techniques and advances in computational methods for language analysis to map opinion landscapes on cases such as migration, antisemitism, antagonistic right-wing discourse, and climate change. These methods, resulting from the project’s fundamental research, are made openly available in an ecosystem of tools and techniques for computational social science called Penelope (Penelope, 2019a; Willaert et al., 2020). The Penelope ecosystem thus comprises modules (individual data-analytical components, often without a user interface) and observatories, which chain together components and have a user interface that allows for the exploration of a specific theme, such as language propagation on social media (Willaert et al., 2021).

Of particular interest to the challenge of constructing reading instruments for online news, is the project’s previous work on a tool called the *Penelope climate change opinion observatory* available at https://penelope.vub.be/observatories/climate-change-opinion-observatory/. This tool taps into a range of data sources, including articles from the leading news website *The Guardian*, as well as a series of experimental modules grounded in the project’s methodological research, in order to provide insights into different aspects of debates on climate change. A key analytical module of the observatory is the Penelope semantic frame extractor, which is based on the ODYCCEUS project’s fundamental research in the field of computational analysis of language. This linguistic research, which also forms the main scientific background for the present paper, can be situated in the field of argument and opinion mining. This field seeks to develop methods for identifying and extracting structures of argumentation and inferencing expressed in natural language (Lawrence and Reed, 2020). Situated among on-going work on the automated detection of arguments and opinions about climate change (Alashri et al., 2016), the ODYCCEUS project’s approach to mining argumentative statements focuses on semantic frame extraction: a natural language understanding task that consists in identifying all instances of selected semantic frames in a given text. In linguistic theory, semantic frames are defined as coherent structures of related concepts. Instances of the “causation” frame, such as “climate change causes global warming,” can thereby be understood as a relation between a “cause” frame element (“climate change”) and an associated “effect” frame element (“global warming”), triggered by a frame-evoking element (“causes”). Semantic frames thus make for form-meaning mappings, many of which are documented and defined in knowledge bases such as the FrameNet project (Baker et al., 1998) or PropBank (Palmer et al., 2005). Building on recent advances in the field, this article operationalizes a novel approach for extracting semantic frame instances from texts that is elaborated in Beuls et al. (2021). The latter approach is grounded in computational construction grammar, whereby the frame-semantic structure of a sentence is retrieved through the intermediary of its morpho-syntactic structure (*Technical Implementation* Section). As such, this method for semantic frame extraction yields state-of-the art results without the need for (expensive) annotated data. Applied to a dataset of English newspaper articles on climate change similar to the one that will be central to the present paper, the computational construction grammar approach achieves a word-level F1 score of 78.5%, outperforming a commonly used approach based on conditional random fields (CRFs). As this approach offers a stable method for extracting instances of semantic frames, in particular instances of the causation frame, it has previously been used to analyze belief systems expressed in online news environments, such as the argumentative domains surrounding energy technologies expressed in comments on the news website of *The Guardian* (Willaert et al., 2020; Willaert et al., Under review)^1^.

These previous research outcomes raise the further question of how instances of semantic frames might be used as a means of comparing and interlinking news articles, and to thus act as a basis for systems that expose readers to alternative beliefs and argumentative statements. In order to explore this conceptual transition from opinion *observation* to opinion *facilitation*, the present paper introduces the *Penelope opinion facilitator*: a novel, proof-of-concept reading instrument for online news media that was developed by the authors and that is made available online at https://penelope.vub.be/opinion-facilitator/. This instrument operationalizes methods for the computational analysis of cultural conflict elaborated in the context of the H2020 ODYCCEUS project. This paper thus serves a double objective. For one thing, it showcases an application of the methods and technologies for linguistic analysis from the Penelope ecosystem of tools and techniques for computational social science that are grounded in research on semantic frame extraction. On a technical level, the paper discusses the design choices behind each module, and it will be shown how these can be combined into an instrument that complements the reading experience of the news website *The Guardian* by automatically interlinking news articles on the level of linguistic expression, viz. instances of semantic frames. For another, the paper explores how such an instrument might reconfigure our readings of climate news coverage, based on the case of climate change controversies. Finally, we relate our findings to the future development of computational social science, and discuss pathways for the evaluation of the instrument within the appropriate scientific frameworks, as well as for the future upscaling of qualitative analyses using semantic frames.

## Materials and Methods

### Goal and Concept of the Reading Instrument

The objective of the proposed reading instrument is to maximize the context of news articles on topics associated with cultural conflict, so that readers are exposed to a diverse range of perspectives and opinions about the issue at hand. Concretely, our instrument is constructed in such a way that it extends the reading experience of articles about climate change on the leading news website *The Guardian.* Like many online newspapers, the website of *The Guardian* provides context for its articles by hyperlinking texts to related content on the site, and by adding topic keywords that guide the reader to other potentially relevant articles tagged for that subject. While these are effective means of curating and navigating large collections of articles, they arguably operate on a high level of granularity that targets topics and domains rather than argumentative statements or beliefs *about* those topics. In the case of climate change, this for instance means that readers can find relevant articles by navigating to the “environment” and “climate change” subsections of the main news page, and that at the bottom of each article they can navigate to other articles about mentioned topics, such as “fossil fuels” or “energy.” Certain phrases in an article, for instance “new coalmine,” might be linked to an article discussing news about the installation of a specific new coalmine, thus establishing a broader frame of reference. However, the actual argumentative statements or beliefs in the article texts are not highlighted. The typical architecture and reading experience of a news website thus raises questions about how to support and facilitate the reader’s process of opinion-formation in a more fine-grained manner.

There are many ways in which argumentative statements or beliefs might be expressed linguistically in a news article, and within the scope of this paper the main focus will be on expressions of causation. It can be argued that such statements about the relation between cause and effect are central to the climate change debate, which is for instance marked by conflict and controversy over human responsibility for climate change (e.g., natural vs. man-made global warming). Automatically extracting and linking such expressions might therefore provide key information that can help readers make up their minds about the core issues of the climate change debate. Users of the instrument are thus enabled to browse through texts on climate change based on shared linguistic patterns between those texts, identified on the basis of semantic frame extraction and basic text similarity measures. This means that the tool adheres to a soft interpretation of opinion “facilitation,” that is, texts are presented to the reader in a manner that does not entail any top-down value judgement. The eventual judgment or meaning-making process is situated entirely with the end-user, who is thus confronted with argumentative statements that can confirm or contradict the beliefs that they might hold.

**FIGURE 1:**
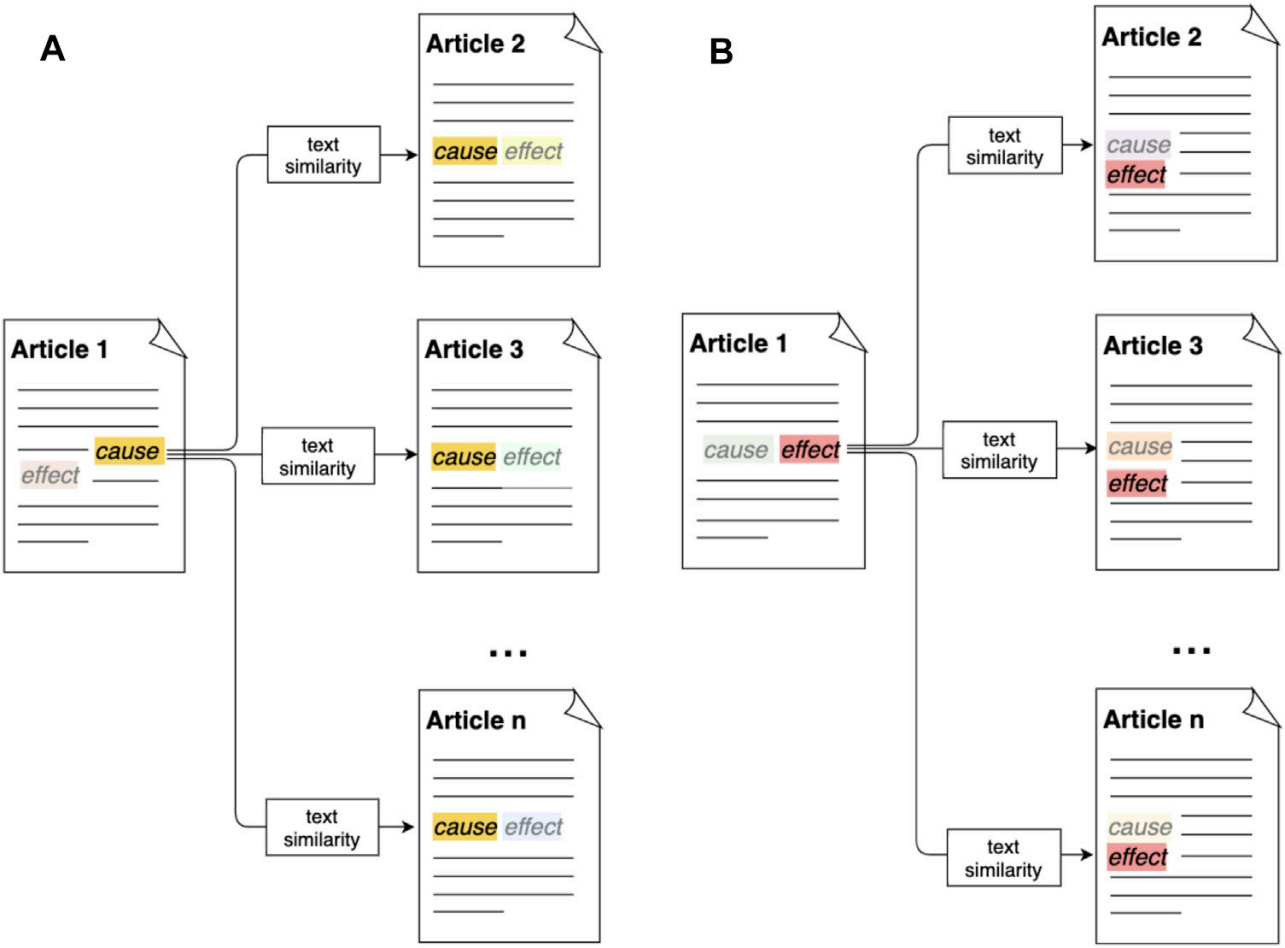
Main working principle of the Penelope opinion facilitator. First, causation frame instances consisting of a “cause” frame element and an “effect” frame element are automatically extracted from news articles on climate using the Penelope semantic frame extractor. Figure 1A **(A)** shows how, subsequently, for a given “cause” frame element, the system is able to retrieve articles expressing alternative “effect” frame elements based on measures for text similarity. Figure 1B **(B)** illustrates how, conversely, the system can retrieve articles containing alternative “cause” frame elements for a given “effect” frame element. Through this mechanism, the system exposes a reader to alternative perspectives on the same issue.

The main working mechanism behind the instrument is to expose the reader to a range of argumentative statements by presenting alternative causes or alternative effects for a given causation frame. As illustrated by the diagrams in Figure 1, instances of the causation frame are automatically extracted from a given Article 1 using the Penelope semantic frame extractor. This comprises a relation between a cause frame element and an effect frame element. For a given “cause” frame element in this article, the user is thus able to explore articles that present alternative “effect” frame elements for this cause frame element (Figure 1A). Conversely, the user can start from a certain “effect” frame element, and explore articles that contain alternative “cause” frame elements for this effect (Figure 1B). Starting for example from the statement “climate change causes global warming,” the tool will thus show the full text of articles mentioning alternative causes for global warming, or articles discussing alternative effects for climate change. This allows the reader to explore alternative argumentative statements, and read the full contexts in which they appear. As such, the instrument can re-contextualize and potentially even change some of the assumptions that the reader might hold.

**FIGURE 2:**
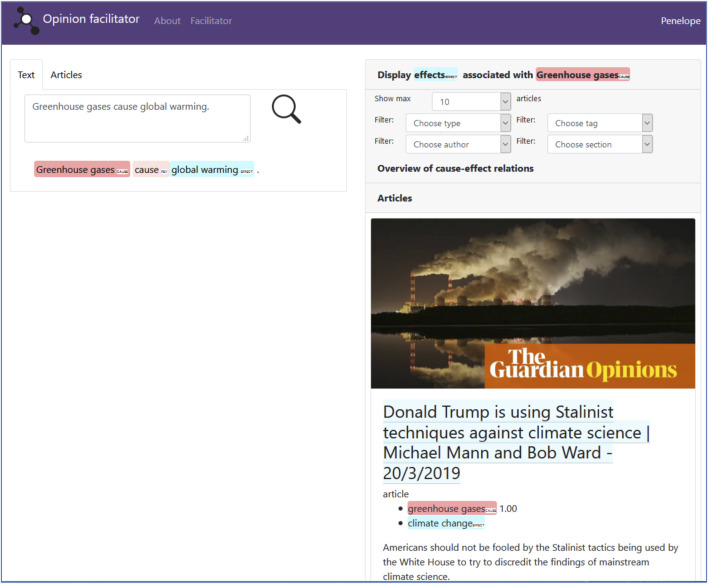
Screenshot of the Penelope opinion facilitator’s user interface. In the left-hand section of the screen, users can enter a causal statement or browse articles. In both cases, “cause” and “effect” frame elements are automatically highlighted in the text, and serve as a basis for further comparison with other articles. In this case, the reader has clicked on the “cause” frame element “greenhouse gases” in the statement “Greenhouse gases cause global warming.” The system then displays articles that mention articles containing alternative “effect” frame elements associated with the “cause” frame element “greenhouse gases.” The suggested articles are ranked on the basis of the similarity between the provided “cause” frame element and the retrieved “cause” frame elements in the articles. Users can read the full texts of the articles and have the option of filtering by a. o. author names in order to explore alternative perspectives on the issue at hand.

Figure 2 shows the workings of this mechanism in the actual tool’s interface. The interface consists of two main sections. In the left-hand section, the user can enter a query in the form of a causal statement, or search the article database and automatically extract instances of the causation frame from those. On the right hand-side, articles containing similar frame elements (causes or effects) for a selected statement are shown. These articles are ranked by the similarity of the frame instances. The menu on top of this right-hand section allows for further filtering, and for displaying the network of associated causes and effects extracted from the articles, with node sizes proportional to the frequency of the retrieved frame elements. As part of the Penelope ecosystem of tools, this reading and browsing instrument makes extensive use of the Penelope Guardian climate dataset and semantic frame extractor modules (“*Technical implementation*”). Yet while the reading tool shares some Penelope modules with the observatory, there are important conceptual and practical differences between both instruments. For one thing, the facilitator targets a more general news readership, rather than the observatory’s audience of researchers. For another, the facilitator is primarily a reading and browsing tool aimed at exposing users to a wide range of argumentative statements, which are shown in the context of the full text, rather than offering the specialized data processing and analysis of the observatory.

### Technical Implementation

**FIGURE 3:**
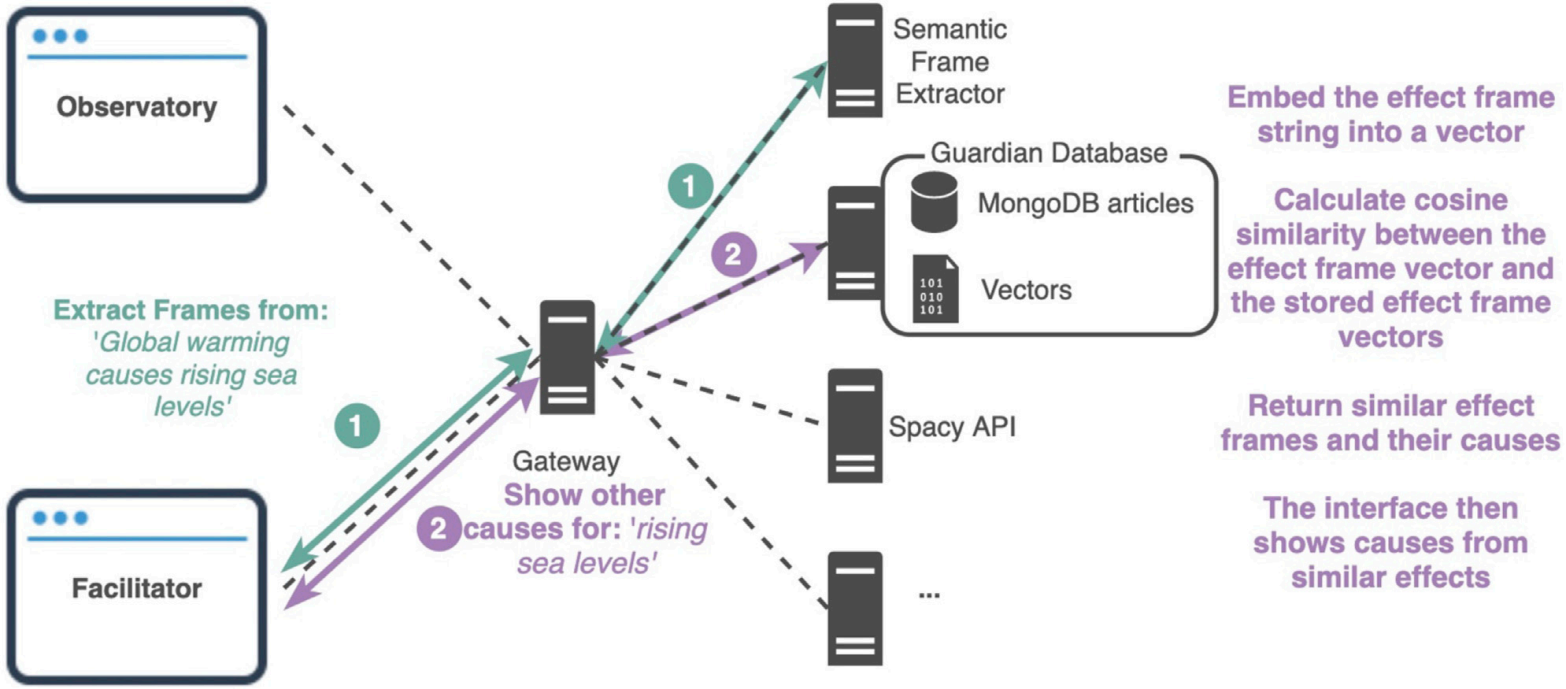
Penelope opinion facilitator and climate change opinion observatory technical diagram. The facilitator system consists of several stand-alone restful web services (or components), which are designed as part of the Penelope ecosystem of tools and techniques for computational social science. The facilitator web application calls the necessary components. The user provides input text and sends a request 1) to the component necessary to extract its causation frames. Consequently, a user can now click on one of the extracted frame instances which will send another request 2) to the database web service, where the embedding of the statement will be compared with the already computed ones in the database. The web service responds with the articles that contain the highest matching causal statements.

The Penelope opinion facilitator was constructed on the basis of the following technical steps (Figure 3):

1. *Data collection:* the corpus of texts at the center of the opinion facilitator is a dataset of articles on climate change from *The Guardian* available from the Penelope ecosystem (Penelope, 2019b). This corpus comprises 12,265 articles from the “Environment” section of the newspaper dated between 2008-09-18 and 2019-04-25.
2. *Semantic frame extraction:* semantic frames (viz. causation frame instances) are extracted from the corpus of texts using the aforementioned computational construction grammar approach to semantic frame extraction (Beuls et al. 2021). The semantic frame extractor that operationalizes this method is openly available from the Penelope ecosystem of tools as a web service. When presented with a text, the frame extractor first splits the texts into sentences and builds a dependency structure of those sentences using the SpaCy natural language processing library. Next, grammatical constructions that encode expert linguistic knowledge enrich the dependency structure with information about semantic frames and their frame elements. This mapping between the morpho-syntactic structure of the sentence and the linguistic knowledge about the causation frames is achieved through a grammar of constructions written in Fluid Construction Grammar (Steels, 2011). The system thereby first seeks to apply constructions that consist of the lexical units (verbs, prepositions and conjunctions) listed in FrameNet as frame evoking elements of the “causation” frame: “cause,” “due to,” “because,” “because of,” “give rise to,” “lead to,” and “result in” (FrameNet, n.d.). When such a construction applies, further constructions are applied that map between the argument structure of the sentence and the “cause” and “effect” frame elements of the causation frame. Finally, the extracted semantic frame instances are added as a field to the articles in the database. Specifically, for each causation frame instance that is extracted from the text, we store the strings that correspond to the “cause” frame element (if any), the “effect” frame element (if any), and the frame evoking element.
3. *Frame element embeddings*: A matrix with the embeddings of the strings of the extracted frame elements (causes or effects) is created using the Python scikit-learn library. We thereby first train a frequency-based embedding model by passing the list of frame element strings (causes or effects) to the CountVectorizer fit function. Then, this model is transformed into a matrix with the embedding for each frame element. These vectors are stored together with the corresponding article-IDs in a separate database. When a user enters a query sentence as string, the embeddings of the frame elements in this query string are obtained using the same procedure.
4. *Text similarity*: as a measure of text similarity, the cosine distance between the embeddings of the frame element in the query string and those of the relevant frame element strings in the articles is calculated. For a given causal statement extracted from an article or provided as a query by the user, suggested articles are ranked based on the similarity of the frame instances they contain in relation to the provided frame instance. Frames with a cosine distance over 0.5 are not presented to the user, as these would be considered too different to have a meaningful relation with the initial query.
5. *Interface:* the interface presents the full texts of the articles around the retrieved frame instances in order to maximize information context. Further filtering options based on article metadata such as keywords, author and newspaper section are provided. The interface is situated within the design space of the Penelope open tools for media observation.

## Results: Use Case on Global Warming Controversies

The Penelope opinion facilitator complements the reading experience of the news website of *The Guardian* by means of an interface for navigating an alternative organization of news articles (based on the similarity of the causation frame instances they contain), with the aim of reconfiguring the ways in which articles on the topic of climate change can be read. By means of a use case on global warming controversies, the present section will demonstrate how the interface exposes alternative beliefs to the one held or tested by the reader, thus supporting the process of opinion-formation and debate facilitation.

As one of the defining issues of our time, the debate surrounding climate change is heavily marked by questions of causation and responsibility. Opinions might for instance clash over the potential beneficial or adversarial ecological effects of different energy sources, such as fossil fuels or renewables (ProCon, 2020a). On a more fundamental level, the climate change debate might also concern diverging assessments of whether global warming is man-made or not (ProCon, 2020b). The question then is how someone who is for instance convinced that global warming is the result of natural causes, might also be exposed to alternative positions, for instance in news articles that discuss human responsibility and man-made causes of global warming.

**FIGURE 4:**
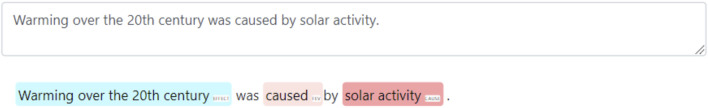
Detail of the Penelope opinion facilitator’s user query window. In this case, the reader entered the statement “Warming over the 20th century was caused by solar activity.” The semantic frame extractor then identifies the “cause” frame element “Warming over the 20th century,” the frame evoking element “caused,” and the “effect” frame element “solar activity.” These frame elements can then be used as the start of a search process for alternative causes and effects.

From a reader’s perspective, one way to use the opinion facilitator is to start from a user query in the form of a causal statement, that is, a certain causal belief that the reader of the newspaper might hold. As shown in Figure 4, an example of a rather controversial statement might be one in which global warming is fully attributed to natural causes, such as “Warming over the 20th century was caused by solar activity.” When the reader enters this statement into the opinion facilitator’s query field, the “cause” and “effect” frame elements in the statement are automatically highlighted.

**FIGURE 5:**
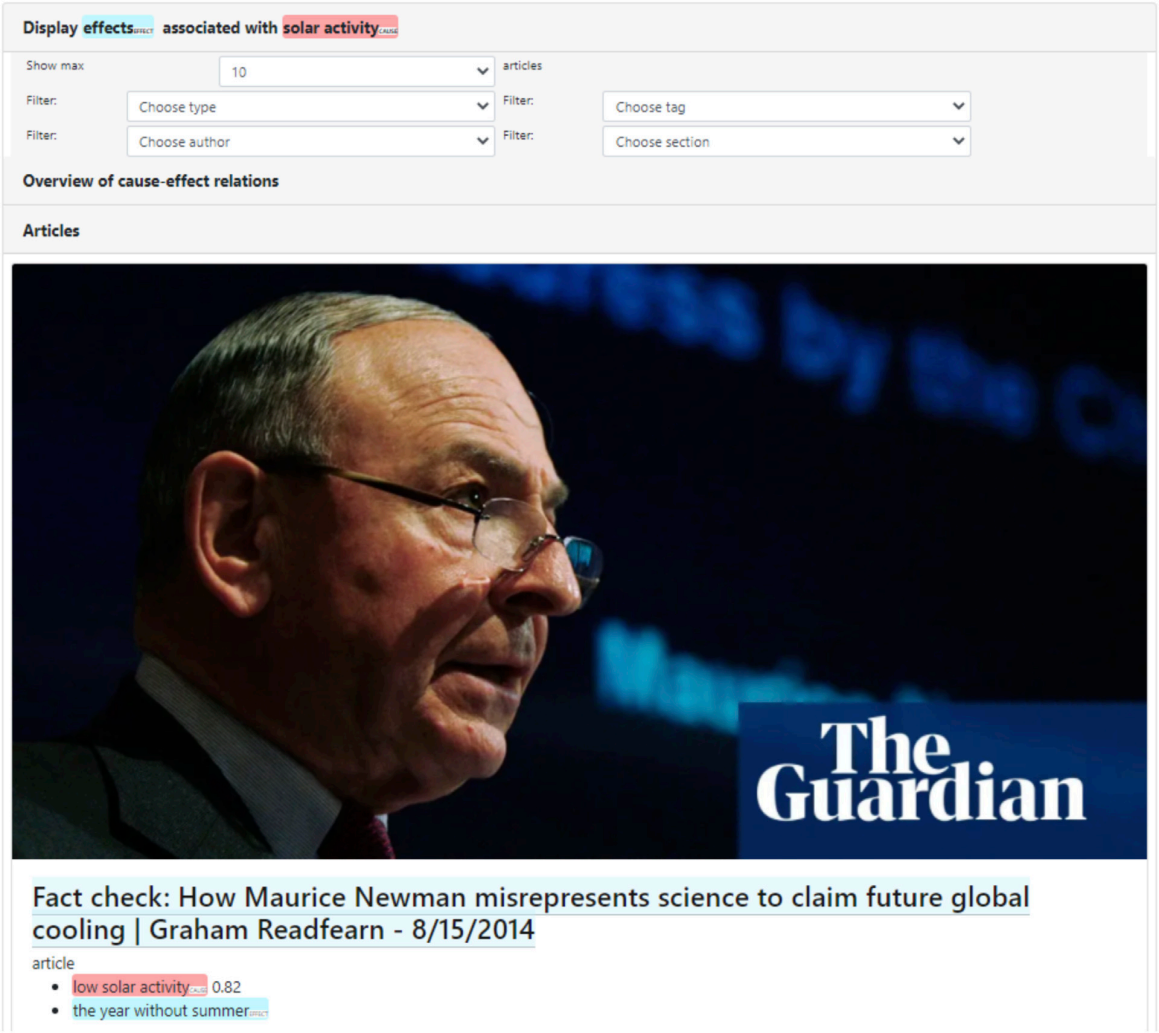
Detail of retrieved articles containing alternative “effect” frame elements for the “cause” frame element “solar activity.” The retrieved article positions debates about the effects of “(low) solar activity” in the context of fact-checking and misrepresentations of science.

In a next step, the user might start contextualizing this statement or belief by examining articles that mention other effects for “solar activity”. This can be done by clicking the effect frame element. The system will then retrieve those articles from the database that include a causation frame instance that contains “solar activity” as “cause” frame element, and any (unspecified) “effect” frame element. As can be seen in Figure 5, other articles that discuss the effects of “increased solar activity” (or similar frame elements) in the context of the climate change debate, thus tend to be about debunking disinformation or misinformation, with titles such as “Fact check: How Maurice Newman misrepresents science to claim future global cooling” (2014), or “Boris Johnson says snow casts doubt on climate change science” (2013). In this specific case, the retrieved articles that are shown in full text in the interface, explicitly contradict the statement provided by the reader, for instance through titles such as “Global warming is not due to the Sun, confirms leaked IPCC report” (2014). Based on the nature and contents of the retrieved articles, the reader who uses the facilitator might thus reconsider the validity of their initial causal statement, and explore arguments in favor of anthropogenic global warming.

**FIGURE 6:**
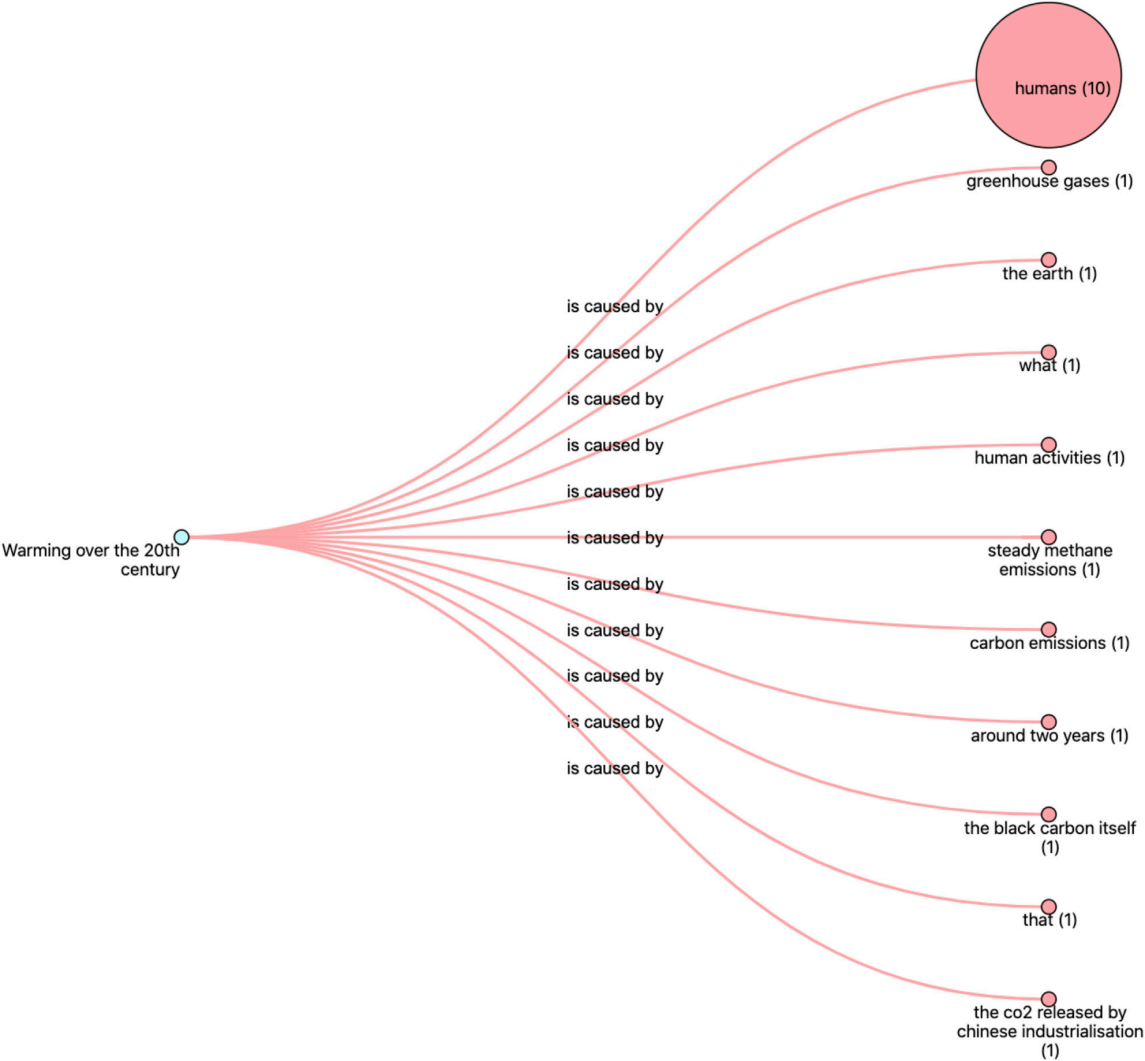
Detail of the Penelope opinion facilitator’s graph overview of retrieved frames. The node on the left represents the selected effect “Warming over the 20th century,” the nodes on the right represent the associated “cause” frame elements retrieved from the articles (for 20 articles). The node sizes are proportional to the frequency of the retrieved frame elements. From this representation, it is clear that “humans” are most frequently mentioned as the cause of “warming over the 20th century.”

As a follow-up to their initial query for articles discussing alternative effects of “solar activity,” the reader might then explore articles that discuss alternative causes associated with “warming over the 20th century.” Following a similar approach, it is possible to click the initial statement’s “effect” frame element in the interface to retrieve these alternative causes. In this case, using the interface’s graph representation option, the reader can explore a networked view of the retrieved cause-effect relations (Figure 6). In this representation, the size of the nodes is proportional to the frequency of the retrieved frame instance. For the example at hand, it thus becomes increasingly obvious that “humans” are a frequently mentioned cause of global warming. Browsing through the retrieved articles reveals titles such as “Survey finds 97% climate science papers agree warming is man-made” (2016), and articles discussing the specific impacts of (man-made) global warming, such as “Climate change is increasing flood risks in Europe” (2018). The reader can balance this new set of observations opened by the tool with his previous explorations, and potentially shift their opinion towards the idea that global warming indeed is not necessarily caused by fluctuations in solar activity, but rather that it is a man-made (anthropogenic) phenomenon. This case example thus illustrates how the proposed reading instrument achieves its primary object of exposing readers to alternative perspectives and points of view on a given issue.

## Discussion and Future Work

In this paper we have introduced and discussed a proof-of-concept reading instrument for online news websites, aimed at exposing the readership of online news to a diverse range of opinions and perspectives with regards to cases of cultural conflict. It was thereby argued that any such instrument should first and foremostly be grounded in an actual understanding and granular representations of the complexities of societal debates. The reading instrument proposed in this paper therefore uses methods for the computational analysis of language from the H2020 ODYCCEUS project’s Penelope infrastructure of tools and techniques for computational social science. Specifically, the instrument under discussion makes use of components for text analysis initially developed for the observation of aspects of cultural conflict related to the climate change debate. In this manner, this instrument and paper mark a conceptual shift from the development of data-analytics tools for debate observation towards the creation of reading instruments for opinion and debate facilitation that can serve a wide readership of online newspapers. Through the case example of global warming controversies, it has specifically been shown how the method of semantic frame extraction that was originally developed to study expressions of beliefs or opinions in texts, can also be used as a basis for comparing news articles, and to suggest relevant content based on the similarity of actual argumentative statements.

For the target audience of the discussed implementation of the instrument—the readership of online newspapers—our approach distinguishes itself from other methods of content curation and recommendation in that it offers a weak interpretation of debate facilitation, which is based entirely on the similarity of specific semantic frames in texts, and thus operates in a fully bottom-up manner. The instrument thus presents readers with texts expressing alternative beliefs, opening up a wide scope of possibilities to explore, but leaving the final judgment to the reader. The tool thus *complements* rather than replaces the judgement and reading experience of the target audience. Regarding the validity of this approach, this article has mainly focused on the technical aspects of the Penelope opinion facilitator, that is, how an experimental method for semantic frame extraction can be operationalized in an openly available proof-of-concept instrument. The paper has thereby noted and referred to resources concerning the internal validity of the semantic frame extraction method, providing evidence that the method is picking up the range of plausible alternative causes and effects in the data based on metrics for precision and recall (Beuls et al. (2021)). As such, however, the paper only covers part of the ODYCCEUS project’s wider research project into opinion facilitation, which also involves testing and validation of the developed instruments with the wider public and stakeholders such as foundations, media companies, and institutions and companies working in the area of opinion dynamics and conflict management (for a follow-up project, Trendify, n.d.). Future work on the facilitator should therefore address further user testing and validation of the instrument within the appropriate scientific frameworks. Firstly, this concerns evaluating the usability of the software infrastructure. As exemplified in Heyvaert et al. (2018), a potential evaluation method would consist of splitting up participants into two groups, one group with participants who have previous expertise, and one group with participants without previous expertise. Next, one would let both groups complete a series of hands-on task scenarios with two different tools, viz. the opinion facilitator instrument and an established tool that has similar functions, such as the current website of *The Guardian* with its navigation and interlinking functionalities for news articles. This approach, a variation of which was previously used to test the usability of the Penelope infrastructure (Penelope, 2019c), would allow for an evaluation of the new tool in relation to existing alternatives, as well as for a stand-alone test of the instrument itself. Secondly, and on a more fundamental level, additional work is needed to evaluate whether the opinions or beliefs held by users interacting with the system are in fact changed or otherwise impacted. This question could for instance be approached through a long-term longitudinal study whereby the attitudes of participants are being evaluated at different moments in time. Such a study should take into account that any shifting attitudes can never be fully ascribed to the use of the platform, and other parameters that might influence participants’ opinions should be brought to the foreground through the testing’s questionnaire. The design of such a study might therefore be informed by insights on opinion-formation from the field of social cognition (Aronson, 2018, Ch. 2).

Beyond the aforementioned communities of users and stakeholders, this proof-of-concept reading instrument also opens up perspectives for the wider research community, in particular with regard to the development of computational social science and related fields such as the digital humanities. The reading instrument thereby offers a step towards machine-guided forms of reading, and notably brings into view opportunities for automating and upscaling the qualitative process of “close reading”. This move hinges on the automatic extraction of instances of semantic frames, which connect the syntactic information available in texts with a meaning representation (e.g., Abstract Meaning Representation (AMR) or other meaning representations used in projects such as PropBank). While the present demonstrator makes use of only one specific frame (the causation frame), recent advances in the fields of artificial intelligence and computational construction grammar are yielding larger grammars containing form-meaning mappings based on substantial annotated corpora of PropBank frames. Such large grammars enable the extraction of semantic frames that go well beyond causation, and open up future avenues of research that include querying corpora of texts based on meaning representations rather than string matching, as well as qualitative discourse analysis at larger scales by automatically identifying *any* frame present in a text. The latter thereby holds the promise of allowing researchers in the social sciences and humanities to assign a greater role to computational methods in the process of assigning meaning to textual data.

## Data Availability

The Penelope opinion facilitator introduced in this paper is available at https://penelope.vub.be/opinion-facilitator/. The climate change news article dataset that was used for this project can be accessed through the Penelope ecosystem of tools and techniques for computational social science at https://penelope.vub.be/components/.

## Footnotes

1 Willaert, T., Banisch, S., Van Eecke, P., Beuls, K. Tracking Causal Relations in the News: Data, Tools, and Models for the Analysis of Argumentative Statements in Online media. Under review.
